# Impact of gestational diabetes mellitus and hypertensive disorders of pregnancy on adverse pregnancy outcomes: a retrospective cohort study

**DOI:** 10.3389/fendo.2026.1805525

**Published:** 2026-05-13

**Authors:** Junjia Shao, Xia Li, Lifeng Wang, Bo Dong

**Affiliations:** Department of Obstetrical, Dongyang Maternal and Child Health Hospital, Jinhua, Zhejiang, China

**Keywords:** adverse pregnancy outcomes, gestational diabetes mellitus, hypertensive disorders of pregnancy, interaction, restricted cubic spline, retrospective cohort study

## Abstract

**Objective:**

To investigate the independent and joint effects of gestational diabetes mellitus (GDM) and hypertensive disorders of pregnancy (HDP) on adverse pregnancy outcomes, evaluate their potential interaction, and explore the nonlinear dose–response relationships between blood glucose and blood pressure levels and composite adverse outcomes using a restricted cubic spline (RCS) model.

**Methods:**

This retrospective cohort study included 240 women who delivered at Dongyang Maternal and Child Health Hospital between January 2020 and December 2024. Participants were categorized into four groups according to the presence of GDM and/or HDP: control, GDM only, HDP only, and comorbid GDM + HDP. The primary outcome was a composite of adverse pregnancy outcomes, including preterm birth, Apgar score < 7, neonatal intensive care unit (NICU) admission, and fetal growth abnormalities. Multivariable logistic regression models were applied to estimate adjusted odds ratios (aORs) and 95% confidence intervals (CIs). Additive interaction indices—relative excess risk due to interaction (RERI) and attributable proportion (AP)—were calculated to assess synergistic effects. Nonlinear dose–response associations between mean fasting glucose, systolic blood pressure (SBP).

**Results:**

Compared with the control group, HDP was independently associated with a higher risk of composite adverse outcomes (aOR = 3.30, 95% CI: 1.41–7.70, P = 0.006), while comorbid GDM + HDP showed a substantially elevated risk (aOR = 9.25, 95% CI: 2.63–32.45, P < 0.001). Additive interaction analysis demonstrated a significant positive interaction between GDM and HDP (RERI = 5.62; AP = 0.61), suggesting that over half of the excess risk was attributable to their joint effect. Specifically, the risks of preterm birth and small-for-gestational-age infants increased markedly in the HDP and comorbid groups, whereas GDM alone was primarily associated with large-for-gestational-age outcomes. RCS analysis revealed a nonlinear positive relationship between mean SBP and composite adverse outcomes, with the risk increasing sharply beyond approximately 135 mmHg and accelerating above 160 mmHg.

**Conclusion:**

Both GDM and HDP independently increase the risk of adverse pregnancy outcomes, and their coexistence exerts a synergistic effect that amplifies maternal and neonatal risk. The nonlinear dose–response pattern between SBP and adverse outcomes highlights that even mild elevations in blood pressure can have detrimental effects.

## Introduction

Hypertensive disorders of pregnancy (HDP) and gestational diabetes mellitus (GDM) are among the most common and preventable pregnancy complications worldwide, imposing substantial short- and long-term health burdens on both mothers and their offspring (1–4). Epidemiological studies estimate that the prevalence of GDM ranges from 5% to 20% depending on diagnostic criteria and population characteristics, with a steady increase following the adoption of the International Association of Diabetes and Pregnancy Study Groups (IADPSG) criteria (1, 5). The global burden of HDP has remained persistently high, and in some regions, its incidence has shown little sign of decline (2, 6). Both disorders are associated not only with adverse perinatal outcomes such as preterm birth, fetal growth abnormalities, and cesarean delivery but also with a heightened risk of postpartum cardiometabolic diseases (4, 7, 8).

Pathophysiologically, GDM and HDP share several underlying mechanisms, including insulin resistance, systemic inflammation, oxidative stress, and endothelial dysfunction, which collectively impair placental perfusion and glycocalyx integrity, leading to placental insufficiency and increased risk of adverse pregnancy outcomes (9–11). Maternal hyperglycemia is closely linked to fetal overgrowth and large-for-gestational-age (LGA) infants, and these conditions have been further associated with an elevated risk of obesity and metabolic disorders later in childhood (3). In contrast, HDP is characterized by placental hypoperfusion, endothelial injury, and suboptimal neonatal adaptation (4, 9).

Of particular concern, the coexistence of GDM and HDP may not merely represent the sum of their individual effects. Recent regional cohort studies have shown that women with comorbid GDM and HDP have a significantly increased risk of preterm birth, fetal growth restriction, and neonatal intensive care unit (NICU) admission compared with those with a single complication, suggesting a potential synergistic interaction between the two (12, 13). From a methodological standpoint, additive interaction measures—such as the relative excess risk due to interaction (RERI) and the attributable proportion (AP)—are valuable for quantifying the excess risk attributable to dual exposure, thereby identifying populations that may benefit from intensified management (14). Furthermore, restricted cubic spline (RCS) models allow for a detailed characterization of nonlinear dose–response relationships between continuous exposures (e.g., blood glucose and blood pressure) and pregnancy outcomes, providing a quantitative framework for identifying clinical thresholds and intervention windows (15, 16).

Given this context, the present study aimed to compare pregnancy outcomes among women with GDM, HDP, and comorbid GDM + HDP, to evaluate the potential additive interaction between these conditions, and to use RCS modeling to explore the nonlinear associations of blood glucose and blood pressure levels with composite adverse pregnancy outcomes. The findings are intended to provide evidence for improved risk stratification and individualized perinatal management strategies.

## Materials and methods

### Study design and participants

This single-center retrospective cohort study was conducted at the Department of Obstetrics, Dongyang Maternal and Child Health Hospital, and included 240 women who delivered between January 2020 and December 2024. All participants had singleton pregnancies and complete clinical data available from both the mid-gestational and delivery periods. The study protocol was reviewed and approved by the Ethics Committee of Dongyang Maternal and Child Health Hospital (Approval No.: 2025-LWP-75). Given the retrospective design, patient identifiers were anonymized, and the requirement for written informed consent was waived.

Participants were categorized into four groups according to the presence of gestational diabetes mellitus (GDM) and/or hypertensive disorders of pregnancy (HDP): Control group: Women without GDM or HDP; GDM group: Women diagnosed with GDM only; HDP group: Women diagnosed with HDP only, including gestational hypertension, preeclampsia, and severe preeclampsia; Comorbid group (GDM + HDP): Women diagnosed with both GDM and HDP.

### Inclusion and exclusion criteria

Inclusion criteria: (1) Age between 18 and 45 years; (2) Singleton pregnancy with both prenatal care and delivery completed at this hospital; (3) Diagnosis or exclusion of GDM and HDP after 20 weeks of gestation; (4) Availability of complete clinical, laboratory, and obstetric outcome data; (5) Live-born fetus without major congenital malformations, defined as clinically significant structural or chromosomal anomalies diagnosed prenatally or at birth that may affect perinatal outcomes.

Exclusion criteria: (1) Pre-existing diabetes mellitus or chronic hypertension; (2) Severe hepatic, renal, cardiovascular, or thyroid diseases; (3) Autoimmune disorders (e.g., systemic lupus erythematosus, antiphospholipid syndrome); (4) Multiple pregnancy or fetal chromosomal/structural abnormalities; (5) Malignancy, infectious disease, or drug-induced metabolic disorders; (6) Incomplete medical records or loss to follow-up.

### Diagnostic criteria

#### Gestational diabetes mellitus

Diagnosis was based on the Guidelines for Diagnosis and Management of Gestational Diabetes Mellitus (China, 2020). All participants underwent a 75 g oral glucose tolerance test (OGTT) at 24–28 weeks of gestation. GDM was diagnosed if one or more of the following plasma glucose thresholds were met or exceeded: (1)Fasting ≥ 5.1 mmol/L; (2)1-hour ≥ 10.0 mmol/L, (3)2-hour ≥ 8.5 mmol/L.

#### Hypertensive disorders of pregnancy

Diagnosis followed the Guidelines for Diagnosis and Treatment of Hypertensive Disorders in Pregnancy (China, 2020). Gestational hypertension was defined as new-onset systolic blood pressure (SBP) ≥ 140 mmHg and/or diastolic blood pressure (DBP) ≥ 90 mmHg after 20 weeks of gestation in previously normotensive women. Preeclampsia was defined as gestational hypertension accompanied by proteinuria (≥ 0.3 g/24 h) and/or multisystem involvement. Severe preeclampsia was defined as preeclampsia accompanied by severe hypertension and/or evidence of significant maternal organ dysfunction, placental dysfunction, or fetal compromise, indicating a substantial risk to the mother or fetus.

### Data collection and variable definitions

Clinical data were retrieved from the hospital’s electronic medical record system, including comprehensive information on maternal demographics, laboratory findings, pregnancy and delivery characteristics, and maternal and neonatal outcomes. The collected variables included maternal age (years), pre-pregnancy body mass index (BMI, kg/m²), parity (≥1 delivery), smoking history, and the use of assisted reproductive technology (ART). Laboratory and physiological parameters comprised fasting, 1-hour, and 2-hour plasma glucose levels from the oral glucose tolerance test (OGTT), mean fasting plasma glucose (mmol/L), mean systolic blood pressure (SBP, mmHg), and mean diastolic blood pressure (DBP, mmHg). Blood pressure was measured by trained nurses using a calibrated mercury sphygmomanometer, and the mean value was calculated from multiple readings obtained during the second and third trimesters and at delivery. Pregnancy and delivery characteristics included gestational age at delivery (weeks), postpartum hemorrhage volume (mL), neonatal birth weight (g), and neonatal length of hospital stay (days). The mode of delivery was categorized as vaginal or cesarean section. The type of hypertensive disorder of pregnancy (HDP) was classified according to diagnostic criteria as none, gestational hypertension, preeclampsia, or severe preeclampsia. In addition, information was recorded regarding the use of aspirin, insulin, and antihypertensive medications during pregnancy.

### Outcome measures

Primary composite adverse outcome:

Defined as the occurrence of any of the following clinically significant perinatal complications: preterm birth (< 37 weeks), Apgar score < 7, neonatal intensive care unit (NICU) admission, or fetal growth abnormality, including small-for-gestational-age (SGA) or large-for-gestational-age (LGA) infant. This composite outcome was used to reflect the overall burden of adverse perinatal outcomes associated with maternal metabolic and hypertensive disorders and to improve statistical efficiency in the setting of a modest sample size.

Secondary outcomes:

Included preterm birth, placental abruption, severe postpartum hemorrhage (≥ 1000 mL), maternal intensive care unit (ICU) admission, large-for-gestational-age (LGA) infant, SGA infant, neonatal hypoglycemia, neonatal respiratory distress, Apgar score < 7, and NICU admission.

### Statistical analysis

All analyses were performed using SPSS version 26.0 (IBM Corp., USA) and R version 4.3.2 (R Foundation for Statistical Computing, Vienna, Austria). Continuous variables with normal distributions were expressed as mean ± standard deviation (SD) and compared using one-way analysis of variance (ANOVA). Non-normally distributed variables were expressed as median (interquartile range) and compared using the Kruskal–Wallis test. Categorical variables were presented as counts and percentages and compared using the χ² test or Fisher’s exact test where appropriate.

Multivariable logistic regression models were applied to evaluate the independent associations of GDM and HDP with adverse pregnancy outcomes, with adjusted odds ratios (aORs) and 95% confidence intervals (CIs) calculated after controlling for potential confounders (age, BMI, parity, and assisted reproduction). Additive interaction between GDM and HDP was assessed by calculating the relative excess risk due to interaction (RERI) and attributable proportion (AP).

**Figure 1:**
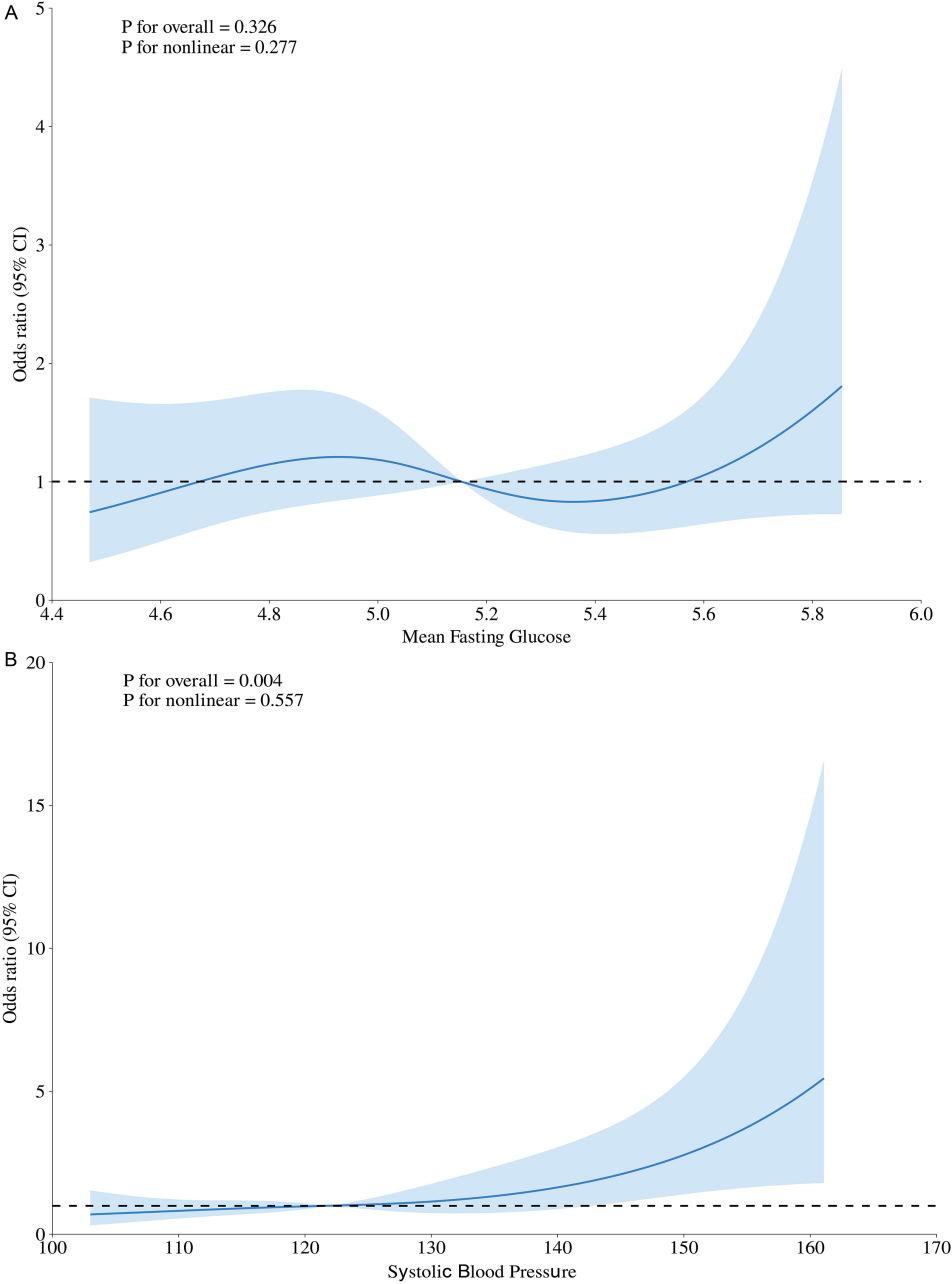
Restricted cubic spline curves for random blood glucose and mean systolic blood pressure with composite outcomes. **(A)** Mean Fasting Blood Glucose; **(B)** Mean Systolic Blood Pressure OR was adjusted for Age、BMI、Parity (≥ 1 Delivery)、Assisted Reproductive Technology and Smoking.

Restricted cubic spline (RCS) regression was further used to examine nonlinear dose–response relationships between blood glucose and blood pressure levels and the risk of composite adverse outcomes. Results were illustrated using spline plots (**Figure 1**).

## Results

### Baseline characteristics

The baseline characteristics and pregnancy outcomes of the study population are presented in **Table 1**. A total of 240 pregnant women were included, comprising 114 in the control group, 58 in the gestational diabetes mellitus (GDM) group, 38 in the hypertensive disorders of pregnancy (HDP) group, and 30 in the comorbid GDM + HDP group. There were no significant differences among the four groups in maternal age, body mass index (BMI), parity, smoking status, or history of assisted reproduction (all P > 0.05).

**Table 1:** Comparison of baseline characteristics and pregnancy-related outcomes in populations with gestational hypertension, diabetes mellitus, and comorbidity of both.

Variables	None (n = 114)	Gestational diabetes mellitus (n = 58)	Hypertensive disorders in pregnancy (n = 38)	Comorbidity (n = 30)	P
Age (years), Mean ± SD	32.06 ± 4.76	31.71 ± 4.79	31.36 ± 4.94	32.02 ± 4.40	0.87
BMI (kg/m²), Mean ± SD	24.77 ± 3.80	23.60 ± 3.51	23.86 ± 3.46	23.94 ± 3.15	0.178
OGTT Fasting Blood Glucose (mmol/L), M (Q_1_, Q_3_)	4.72 (4.45,4.88)	5.53 (5.33,5.89)	4.77 (4.53,4.98)	5.58 (5.32,5.70)	<0.001
OGTT 1-Hour Blood Glucose (mmol/L), M (Q_1_, Q_3_)	8.20 (7.78,8.75)	10.93 (10.26,11.80)	8.51 (7.98,8.86)	10.91 (10.14,11.40)	<0.001
OGTT 2-Hour Blood Glucose (mmol/L), M (Q_1_, Q_3_)	6.62 (6.15,7.14)	9.30 (8.75,9.90)	6.75 (6.22,7.34)	9.49 (9.02,9.93)	<0.001
Mean Fasting Blood Glucose (mmol/L), M (Q_1_, Q_3_)	5.06 (4.80,5.24)	5.46 (5.29,5.66)	5.02 (4.79,5.17)	5.46 (5.12,5.71)	<0.001
Mean Systolic Blood Pressure (mmHg), M (Q_1_, Q_3_)	116.50 (110.25,122.75)	119.00 (108.25,124.00)	150.00 (143.00,155.75)	150.50 (142.75,158.00)	<0.001
Mean Diastolic Blood Pressure (mmHg), M (Q_1_, Q_3_)	74.00 (68.25,78.00)	73.00 (68.00,76.00)	94.00 (87.25,97.75)	91.00 (87.00,98.75)	<0.001
Gestational Age Weeks, M (Q_1_, Q_3_)	39.20 (38.32,39.88)	38.40 (38.00,39.00)	38.50 (37.60,38.98)	37.30 (36.55,38.10)	<0.001
Postpartum Hemorrhage (ml), M (Q_1_, Q_3_)	501.50 (304.50,722.25)	499.00 (325.75,692.25)	623.00 (435.25,771.50)	721.00 (590.25,834.75)	<0.001
Birth Weight (g), M (Q_1_, Q_3_)	3403.00 (3078.75,3670.00)	3442.00 (3131.25,3731.75)	2964.00 (2795.00,3268.25)	2916.50 (2494.00,3170.00)	<0.001
Neonatal Length of Stay (Days), M (Q_1_, Q_3_)	3.00 (2.00,4.00)	2.00 (1.25,4.00)	4.00 (2.00,6.75)	4.50 (3.00,6.00)	<0.001
Parity (≥ 1 Delivery), n(%)					0.366
No	53 (46.49)	34 (58.62)	22 (57.89)	17 (56.67)	
Yes	61 (53.51)	24 (41.38)	16 (42.11)	13 (43.33)	
Assisted Reproductive Technology, n(%)					0.384
No	101 (88.60)	50 (86.21)	32 (84.21)	29 (96.67)	
Yes	13 (11.40)	8 (13.79)	6 (15.79)	1 (3.33)	
Smoking, n(%)					0.545
No	111 (97.37)	55 (94.83)	36 (94.74)	30 (100.00)	
Yes	3 (2.63)	3 (5.17)	2 (5.26)	0 (0.00)	
Hdp Type, n(%)					<0.001
None	114 (100.00)	58 (100.00)	0 (0.00)	0 (0.00)	
Gestational Hypertension	0 (0.00)	0 (0.00)	22 (57.89)	18 (60.00)	
Preeclampsia	0 (0.00)	0 (0.00)	14 (36.84)	11 (36.67)	
Severe preeclampsia	0 (0.00)	0 (0.00)	2 (5.26)	1 (3.33)	
Aspirin Use, n(%)					<0.001
No	63 (55.26)	18 (31.03)	12 (31.58)	5 (16.67)	
Yes	51 (44.74)	40 (68.97)	26 (68.42)	25 (83.33)	
Antihypertensive Medication Use, n(%)					<0.001
No	114 (100.00)	58 (100.00)	17 (44.74)	12 (40.00)	
Yes	0 (0.00)	0 (0.00)	21 (55.26)	18 (60.00)	
Insulin Use, n(%)					<0.001
No	114 (100.00)	30 (51.72)	38 (100.00)	17 (56.67)	
Yes	0 (0.00)	28 (48.28)	0 (0.00)	13 (43.33)	
Preterm Birth, n(%)					<0.001
No	108 (94.74)	56 (96.55)	31 (81.58)	19 (63.33)	
Yes	6 (5.26)	2 (3.45)	7 (18.42)	11 (36.67)	
Mode Of Delivery, n(%)					<0.001
Vaginal	78 (68.42)	28 (48.28)	21 (55.26)	8 (26.67)	
Cesarean	36 (31.58)	30 (51.72)	17 (44.74)	22 (73.33)	
Severe Postpartum Hemorrhage (≥ 1000 ml), n(%)					0.598
No	109 (95.61)	55 (94.83)	37 (97.37)	27 (90.00)	
Yes	5 (4.39)	3 (5.17)	1 (2.63)	3 (10.00)	
Maternal Icu, n(%)					0.149
No	112 (98.25)	58 (100.00)	37 (97.37)	28 (93.33)	
Yes	2 (1.75)	0 (0.00)	1 (2.63)	2 (6.67)	
Placental Abruption, n(%)					0.071
No	114 (100.00)	57 (98.28)	36 (94.74)	29 (96.67)	
Yes	0 (0.00)	1 (1.72)	2 (5.26)	1 (3.33)	
Neonate Sex, n(%)					0.955
No	63 (55.26)	32 (55.17)	20 (52.63)	15 (50.00)	
Yes	51 (44.74)	26 (44.83)	18 (47.37)	15 (50.00)	
Small for Gestational Age, n(%)					<0.001
No	103 (90.35)	55 (94.83)	24 (63.16)	19 (63.33)	
Yes	11 (9.65)	3 (5.17)	14 (36.84)	11 (36.67)	
Large for Gestational Age, n(%)					<0.001
No	103 (90.35)	38 (65.52)	36 (94.74)	25 (83.33)	
Yes	11 (9.65)	20 (34.48)	2 (5.26)	5 (16.67)	
Apgar Score < 7, n(%)					0.013
No	114 (100.00)	58 (100.00)	37 (97.37)	28 (93.33)	
Yes	0 (0.00)	0 (0.00)	1 (2.63)	2 (6.67)	
Neonatal Hypoglycemia, n(%)					0.046
No	106 (92.98)	50 (86.21)	37 (97.37)	24 (80.00)	
Yes	8 (7.02)	8 (13.79)	1 (2.63)	6 (20.00)	
Respiratory Distress, n(%)					0.501
No	109 (95.61)	53 (91.38)	36 (94.74)	27 (90.00)	
Yes	5 (4.39)	5 (8.62)	2 (5.26)	3 (10.00)	
Nicu Admission, n(%)					0.007
No	106 (92.98)	51 (87.93)	31 (81.58)	21 (70.00)	
Yes	8 (7.02)	7 (12.07)	7 (18.42)	9 (30.00)	
Primary Composite Outcome, n(%)					<0.001
No	58 (50.88)	25 (43.10)	9 (23.68)	3 (10.00)	
Yes	56 (49.12)	33 (56.90)	29 (76.32)	27 (90.00)	

Compared with the control group, women in the GDM and comorbid groups exhibited significantly higher fasting, 1-hour, and 2-hour plasma glucose levels on the oral glucose tolerance test (OGTT) (P < 0.001). The mean systolic and diastolic blood pressure levels were significantly higher in both the HDP and comorbid groups than in the other groups (P < 0.001). The comorbid group also had a shorter mean gestational age and a greater volume of postpartum hemorrhage (P < 0.001).

Regarding pregnancy outcomes, the incidence of adverse outcomes was significantly higher in the HDP and comorbid groups compared with the control group. The rates of preterm birth were 18.4% in the HDP group and 36.7% in the comorbid group, both markedly higher than that in the control group (5.3%, P < 0.001). Cesarean section rates were also elevated in the GDM group (51.7%) and comorbid group (73.3%) compared with the control group (31.6%, P < 0.001). Mean neonatal birth weight was significantly lower in the HDP and comorbid groups (2964 g and 2916 g, respectively) than in the control group (3403 g, P < 0.001).

**Figure 2:**
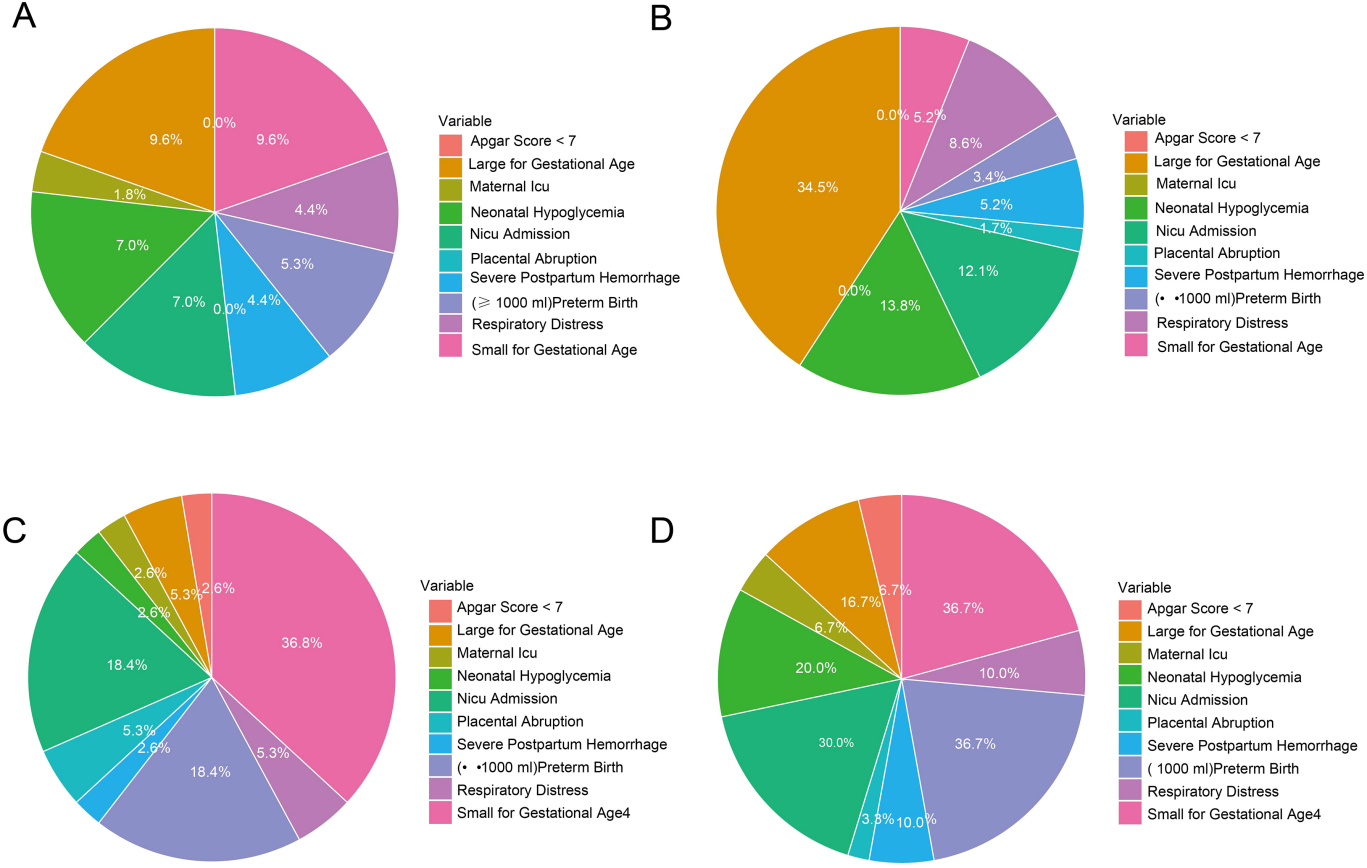
Comparison of differences in pregnancy outcomes among the four groups. **(A)** None; **(B)** Gestational Diabetes Mellitus; **(C)** Hypertensive Disorders in Pregnancy; D:Comorbidity.

In terms of perinatal outcomes, the NICU admission rate was highest in the comorbid group (30.0%), significantly exceeding that of the other three groups (P = 0.007). The proportion of neonates with an Apgar score <7 was higher in both the HDP and comorbid groups (P = 0.013). Furthermore, the incidence of small-for-gestational-age (SGA) infants was significantly increased in the HDP and comorbid groups (36.8% and 36.7%, respectively; P < 0.001), whereas the rate of large-for-gestational-age (LGA) infants was higher in the GDM group (34.5%, P < 0.001). The distribution of adverse pregnancy outcomes across the four groups is illustrated in **Figure 2**.

### Multivariable logistic regression analysis of adverse pregnancy outcomes

Multivariable logistic regression models were constructed with the control group as reference, adjusting for potential confounders such as maternal age, BMI, parity, and assisted reproduction history (**Table 2**). The results showed that HDP significantly increased the risk of composite adverse outcomes—including preterm birth, NICU admission, low Apgar score, and fetal growth restriction (OR = 3.30, 95% CI: 1.41–7.70, P = 0.006). The risk was further elevated among women with both GDM and HDP (OR = 9.25, 95% CI: 2.63–32.45, P < 0.001), indicating a strong synergistic effect between the two conditions.

**Table 2:** The impact of gestational hypertension, diabetes mellitus, and comorbidity on pregnancy outcomes: statistical results and odds ratio analysis.

Variables	β	S.E	Z	P	*OR (95%CI)
Primary composite outcome					
None					1.00 (Reference)
Gestational Diabetes Mellitus	0.28	0.33	0.84	0.4	1.33 (0.69 ~ 2.55)
Hypertensive Disorders in Pregnancy	1.19	0.43	2.76	0.006	3.30 (1.41 ~ 7.70)
Comorbidity	2.22	0.64	3.47	<0.001	9.25 (2.63 ~ 32.45)
Preterm birth					
None					1.00 (Reference)
Gestational Diabetes Mellitus	0.31	0.32	0.96	0.335	1.37 (0.72 ~ 2.58)
Hypertensive Disorders in Pregnancy	1.21	0.43	2.84	0.005	3.34 (1.45 ~ 7.68)
Comorbidity	2.23	0.64	3.51	<0.001	9.32 (2.68 ~ 32.47)
Severe postpartum hemorrhage (≥ 1000 ml)					
None					1.00 (Reference)
Gestational Diabetes Mellitus	0.31	0.32	0.96	0.335	1.37 (0.72 ~ 2.58)
Hypertensive Disorders in Pregnancy	1.21	0.43	2.84	0.005	3.34 (1.45 ~ 7.68)
Comorbidity	2.23	0.64	3.51	<0.001	9.32 (2.68 ~ 32.47)
Maternal icu					
None					1.00 (Reference)
Gestational Diabetes Mellitus	0.31	0.32	0.96	0.335	1.37 (0.72 ~ 2.58)
Hypertensive Disorders in Pregnancy	1.21	0.43	2.84	0.005	3.34 (1.45 ~ 7.68)
Comorbidity	2.23	0.64	3.51	<0.001	9.32 (2.68 ~ 32.47)
Placental abruption					
None					1.00 (Reference)
Gestational Diabetes Mellitus	0.31	0.32	0.96	0.335	1.37 (0.72 ~ 2.58)
Hypertensive Disorders in Pregnancy	1.21	0.43	2.84	0.005	3.34 (1.45 ~ 7.68)
Comorbidity	2.23	0.64	3.51	<0.001	9.32 (2.68 ~ 32.47)
Small for gestational age					
None					1.00 (Reference)
Gestational Diabetes Mellitus	0.31	0.32	0.96	0.335	1.37 (0.72 ~ 2.58)
Hypertensive Disorders in Pregnancy	1.21	0.43	2.84	0.005	3.34 (1.45 ~ 7.68)
Comorbidity	2.23	0.64	3.51	<0.001	9.32 (2.68 ~ 32.47)
Large for gestational age					
None					1.00 (Reference)
Gestational Diabetes Mellitus	1.65	0.44	3.73	<0.001	5.23 (2.19 ~ 12.48)
Hypertensive Disorders in Pregnancy	-0.72	0.81	-0.89	0.372	0.48 (0.10 ~ 2.37)
Comorbidity	0.8	0.6	1.33	0.184	2.22 (0.68 ~ 7.21)
Neonatal hypoglycemia					
None					1.00 (Reference)
Gestational Diabetes Mellitus	0.85	0.54	1.55	0.121	2.33 (0.80 ~ 6.77)
Hypertensive Disorders in Pregnancy	-0.94	1.08	-0.87	0.385	0.39 (0.05 ~ 3.27)
Comorbidity	1.07	0.59	1.81	0.070	2.92 (0.92 ~ 9.30)
Respiratory distress					
None					1.00 (Reference)
Gestational Diabetes Mellitus	0.73	0.67	1.1	0.272	2.08 (0.56 ~ 7.66)
Hypertensive Disorders in Pregnancy	0.23	0.87	0.26	0.792	1.26 (0.23 ~ 6.86)
Comorbidity	0.8	0.77	1.04	0.297	2.22 (0.50 ~ 9.98)
Nicu admission					
None					1.00 (Reference)
Gestational Diabetes Mellitus	0.59	0.55	1.07	0.282	1.81 (0.61 ~ 5.36)
Hypertensive Disorders in Pregnancy	1.09	0.57	1.92	0.055	2.96 (0.98 ~ 8.97)
Comorbidity	1.76	0.55	3.2	0.001	5.83 (1.98 ~ 17.18)

*adjusted for Age、BMI、Parity (≥ 1 Delivery)、Assisted Reproductive Technology and Smoking.

Specifically, the risk of preterm birth was significantly higher in the HDP and comorbid groups (OR = 3.34 and 9.32, respectively; both P < 0.01), suggesting that elevated blood pressure and glucose dysregulation may jointly contribute to placental insufficiency and intrauterine stress, leading to premature termination of pregnancy. GDM was identified as an independent risk factor for large-for-gestational-age (LGA) infants (OR = 5.23, 95% CI: 2.19–12.48, P < 0.001), whereas HDP and comorbidity were associated with increased rates of small-for-gestational-age (SGA) infants (P < 0.001), indicating opposite effects of hyperglycemia and hypertension on fetal growth. In addition, the comorbid group had the highest risk of NICU admission (OR = 5.83, 95% CI: 1.98–17.18, P = 0.001), suggesting that neonates born to mothers with both GDM and HDP were more likely to experience metabolic or respiratory adaptation difficulties, highlighting the need for enhanced perinatal monitoring and early intervention.

### Interaction analysis

To further explore the combined effect of GDM and HDP, an additive interaction model was established (**Table 3**). The results demonstrated a significant positive interaction between GDM and HDP, with the risk of composite adverse outcomes in the comorbid group markedly exceeding the sum of risks associated with each condition alone. The relative excess risk due to interaction (RERI) was 5.62, indicating that the joint exposure produced an additional risk 5.62 times greater than the sum of the individual effects. The attributable proportion (AP) was 0.61, suggesting that approximately 61% of the excess risk could be attributed to the interaction between GDM and HDP.

**Table 3:** Analysis of the interaction between gestational hypertension and gestational diabetes mellitus comorbidity.

Indicator	OR for GDM	OR for HDP	OR for comorbidity	RERI	Attributable proportion	Statistic
Estimated Value	1.33	3.30	9.25	5.62	0.61	3.14

RERI, Relative Excess Risk due to Interaction.

### Dose–response relationship between blood glucose, blood pressure, and adverse outcomes

Restricted cubic spline (RCS) models were used to assess nonlinear dose–response relationships between blood glucose, blood pressure levels, and the risk of composite adverse pregnancy outcomes (**Figure 1**). The association between mean fasting glucose and composite adverse outcomes was not statistically significant (P > 0.05), but the overall trend indicated a positive relationship, implying that higher glucose levels may still contribute to increased risk. Mean systolic blood pressure showed a clear positive and nonlinear association with adverse outcomes, with a progressively steeper rise in risk as systolic pressure increased. The risk began to rise gradually when systolic blood pressure exceeded approximately 120–130 mmHg, became markedly higher beyond 135 mmHg, and accelerated further above 160 mmHg. These findings suggest that even mild elevations in blood pressure may adversely affect maternal and fetal outcomes and that conventional diagnostic thresholds for hypertension in pregnancy might underestimate early risk.

## Discussion

This study systematically analyzed the differences in pregnancy outcomes among women with gestational diabetes mellitus (GDM), hypertensive disorders of pregnancy (HDP), and their coexistence. The results demonstrated that both GDM and HDP independently increased the risk of adverse maternal and neonatal outcomes, whereas their coexistence markedly amplified this risk, exhibiting a clear synergistic and interactive effect. These findings not only confirm the independent impacts of metabolic and hemodynamic abnormalities on perinatal outcomes but also reveal a potential “pathophysiological synergy” when the two conditions overlap.

Consistent with previous studies, our findings showed that women with HDP had significantly higher risks of preterm birth, fetal growth restriction, and neonatal intensive care unit (NICU) admission. These associations may be attributed to impaired placental perfusion and systemic endothelial dysfunction. Previous research has shown that HDP can activate inflammatory pathways and vasoconstrictive mediators, leading to reduced placental blood flow and villous degeneration, which in turn cause chronic fetal hypoxia and nutritional deficiency (17, 18). Meanwhile, the markedly increased incidence of large-for-gestational-age (LGA) infants in the GDM group supports the hypothesis that maternal hyperglycemia promotes excessive fetal growth. Elevated maternal glucose levels facilitate the passive diffusion of glucose across the placenta, stimulating fetal pancreatic β-cell hyperplasia and excessive insulin secretion, which drives fat accumulation and weight gain in the fetus (19, 20).

Of particular note, our study demonstrated that comorbidity of GDM and HDP confers a risk far greater than the sum of their individual effects. Logistic regression analyses revealed that the combined group had significantly higher odds of composite adverse outcomes than either condition alone, and additive interaction analysis (RERI and AP) confirmed a substantial positive interaction between the two. Similar results have been reported in recent multicenter cohort studies (12, 21), suggesting a possible cascade between metabolic disturbances and vascular dysfunction. Hyperglycemia in GDM can induce oxidative stress and endothelial dysfunction, thereby exacerbating hypertension-related vascular damage (10, 22); conversely, placental ischemia caused by HDP may worsen insulin resistance and inflammatory responses, further deteriorating maternal metabolic status. This reciprocal reinforcement between metabolic and vascular abnormalities may represent a key mechanism underlying the sharply increased risk of adverse maternal–fetal outcomes in comorbid cases.

Although the composite outcome improved statistical efficiency, its individual components may represent partially distinct pathophysiological pathways. Therefore, the composite endpoint should be interpreted as a measure of overall perinatal risk burden, whereas the specific associations of GDM and HDP with individual outcomes should be interpreted in light of the separate secondary outcome analyses. In particular, GDM was more strongly associated with LGA, whereas HDP was more strongly associated with preterm birth, SGA, and NICU admission, suggesting heterogeneity across outcome components.

The restricted cubic spline (RCS) model further revealed a nonlinear dose–response relationship between systolic blood pressure (SBP) and composite adverse outcomes. The risk increased progressively with rising SBP and accelerated markedly when SBP exceeded approximately 135 mmHg, with a further steep rise above 160 mmHg. These findings are consistent with recent reports suggesting that even mild elevations in blood pressure may elevate perinatal risk (23, 24). Although the association between fasting glucose and adverse outcomes did not reach statistical significance, a positive trend remained, possibly reflecting effective glycemic management within our cohort (median fasting glucose in the GDM group: 5.46 mmol/L). Importantly, accumulating evidence indicates that maternal glucose levels are continuously associated with perinatal risk even below conventional GDM diagnostic thresholds. The landmark HAPO study, which included more than 23,000 women from nine countries, demonstrated linear associations between maternal OGTT glucose levels and multiple adverse outcomes—including birth weight >P90, cord C-peptide >P90, primary cesarean delivery, and neonatal hypoglycemia—without identifiable thresholds. Subsequent meta-analyses and follow-up studies have reinforced this “metabolic continuum” concept, suggesting that subthreshold hyperglycemia may influence placental nutrient transport, fetal metabolic programming, and long-term offspring outcomes (25, 26).

Clinically, these findings underscore the importance of recognizing comorbid GDM and HDP as a uniquely high-risk condition. Pregnant women affected by both disorders require intensified dual management of glucose and blood pressure, with particular attention to placental function monitoring, serial ultrasound evaluation, and close perinatal surveillance during the mid- to late-gestation period. Recent clinical trials have shown that early interventions—such as low-dose aspirin, balanced diet, and exercise—can effectively reduce the incidence of HDP and its complications (27, 28). Furthermore, postpartum follow-up should not be neglected, as substantial evidence indicates that women with a history of GDM or HDP face significantly increased long-term risks of metabolic syndrome, type 2 diabetes, and cardiovascular disease (7, 29, 30). Therefore, comprehensive perinatal management and long-term cardiometabolic follow-up should be considered essential components of future maternal–fetal health strategies.

Nevertheless, this study has several limitations. First, the retrospective single-center design and modest sample size may introduce selection bias. In particular, the relatively small number of women in the comorbid GDM + HDP group may have limited the statistical power for interaction analyses and reduced the precision of estimates derived from multivariable and spline-based models. Therefore, the interaction effects and nonlinear associations observed in this study should be interpreted cautiously and validated in larger multicenter prospective studies. Second, blood glucose and blood pressure levels were evaluated as average values during mid-to-late pregnancy, without continuous monitoring, potentially underestimating the effects of short-term fluctuations. Third, residual confounding may still exist because several important variables were not fully available for adjustment, including gestational weight gain, socioeconomic factors, dietary habits, pre-pregnancy metabolic disorders, family history of hypertension or diabetes, physical activity, and treatment adherence during pregnancy, such as aspirin dose, antihypertensive medication use, and insulin titration. Finally, most participants with GDM or HDP received medical treatment during pregnancy, which may have mitigated the magnitude of adverse outcomes. Future multicenter prospective studies incorporating metabolomic and placental biomarker analyses are warranted to validate these findings and elucidate the molecular mechanisms and therapeutic targets underlying GDM–HDP comorbidity.

## Conclusion

This study demonstrated that both gestational diabetes mellitus and hypertensive disorders of pregnancy independently increase the risk of adverse pregnancy outcomes, and their coexistence confers a significantly higher, synergistic risk. Systolic blood pressure exhibited a nonlinear dose–response association with composite adverse outcomes, suggesting that even mild elevations may adversely affect maternal and fetal health. Early identification of high-risk women and implementation of dual-targeted interventions for glycemic and blood pressure control may improve perinatal outcomes and reduce long-term cardiometabolic risks in mothers.

## Data Availability

The raw data supporting the conclusions of this article will be made available by the authors, without undue reservation.
